# Association between the estrogen receptor α gene polymorphisms rs2234693 and rs9340799 and severe and mild pre-eclampsia: a meta-analysis

**DOI:** 10.1042/BSR20181548

**Published:** 2019-02-08

**Authors:** Ge Zhao, Yunfei Cai, Jing Liu, Tao Meng

**Affiliations:** 1 Department of Obstetrics, The First Hospital of China Medical University, No. 155 Nanjing Bei Street, Heping District, Shenyang, Liaoning Province, P.R. China 110001; 2 Department of Dermatology, The First Hospital of China Medical University, No. 155 Nanjing Bei Street, Heping District, Shenyang, Liaoning Province, P.R. China 110001

**Keywords:** estrogen receptor alpha, meta-analysis, polymorphisms, preeclampsia

## Abstract

This meta-analysis was performed in order to determine the associations between the estrogen receptor α (ESR1) gene PvuII site (-397T/C, rs2234693) and XbaI site (-351A/G, rs9340799) polymorphisms with severe and mild pre-eclampsia. Eligible studies were identified by searching PubMed, Medline, Embase, China National Knowledge Infrastructure (CNKI), and WanFang databases until May 2018. The pooled odds ratio (OR) and 95% confidence interval (CI) were used to calculate the associations. Six articles (consisting of seven studies; one article was considered as two separate studies with two different subpopulations) investigated the ESR1 gene PvuII -397T/C and XbaI -351A/G polymorphisms in severe and mild pre-eclampsia patients and included controls. The pooled results indicated an increased risk of severe pre-eclampsia for the XbaI -351A/G polymorphism (OR = 1.67, 95% CI = 1.10–2.25, *P*=0.017 for GG compared with AA+GA; OR = 1.81, 95% CI = 1.17–2.82, *P*=0.008 for GG compared with GA). The GG genotype of the ESR1 XbaI polymorphism could be a genetic risk factor for severe pre-eclampsia susceptibility. However, the ESR1 gene PvuII -397T/C polymorphism was not significantly associated with the risk of severe pre-eclampsia, and there was no association between mild pre-eclampsia and the ESR1 gene PvuII -397T/C and XbaI -351A/G polymorphisms separately. The current meta-analysis indicates that the ESR1 XbaI genetic polymorphism may be associated with severe pre-eclampsia. However, there was no association of the ESR1 gene PvuII and XbaI polymorphisms with the risk of mild pre-eclampsia. Owing to the low statistical power, the results may not be sufficiently robust and this conclusion should be interpreted cautiously, which highlights the requirement for large-scale and high-quality studies in this field.

## Introduction

Pre-eclampsia is a major leading cause of maternal mortality worldwide (2–5%), affecting women after 20 weeks of pregnancy, and is characterized by increased systemic vascular resistance, decreased blood volume, vascular endothelial cell destruction, and renal hemodynamic abnormalities [1]. Annually, approximately 40000 maternity patients could die due to pre-eclampsia and eclampsia resulting from a shallow implanted placenta, leading to severe immune reaction involved with inflammatory mediators of the placenta, and acting on the vascular endothelium [1]. Pre-eclampsia is the result of genetic components interacting closely with environmental influences; however, the etiologyof pre-eclampsia remains to be elucidated [2].

The estrogen receptor α (ESR1) gene is located on the long arm of chromosome 6 (6q25.1) and contains eight exons [3]. ESR1 is a ligand-activated transcription factor that can be activated by growth factors in the absence of estrogen [4]. The N-terminus of ESR1 plays a critical role in activating ESR1-reliant genes, and any mutations in this location have been associated with high blood pressure [3,5–7]. Several ESR1 gene polymorphisms have associations with severe and mild pre-eclampsia, and two of the most investigated polymorphisms in intron 1 are ESR1 PvuII -397T/C (rs2234693) and XbaI -351A/G (rs9340799).

The association between ESR1 PvuII -397T/C (rs2234693) and XbaI -351A/G (rs9340799) polymorphisms with the risk of severe and mild pre-eclampsia was found to be inconsistent. Therefore, a meta-analysis of six articles (seven studies) was performed to investigate the association between ESR1 polymorphisms and the risk of severe and mild pre-eclampsia.

## Methods

### Literature search

Literature published until May 2018 was retrieved from the PubMed, Medline, Embase, China National Knowledge Infrastructure (CNKI), and WanFang databases, both English and Chinese language articles were included. The search strategies contained combinations of the following terms: (‘estradiol receptor α’ or ‘ER alpha’ or ‘estrogen receptor 1’ or ‘ESR1’), (‘polymorphism’ or ‘variant’ or ‘mutation’), and (‘pre-eclampsia’ or ‘severe pre-eclampsia’ or ‘mild pre-eclampsia’). Reference lists were also retrieved and manually searched to identify additional potential studies.

### Inclusion and exclusion criteria

#### Inclusion criteria

Included studies met the following criteria: (i) assessing the association of ESR1 -397T/C PvuII (rs2234693) or -351A/G XbaI (rs9340799) polymorphisms with the risk of pre-eclampsia, (ii) sufficient data to calculate the odds ratio (OR) with 95% confidence interval (CI), and (iii) case–control study.

#### Exclusion criteria

Studies were excluded based on the following criteria: (i) overlapping data, (ii) reviews, letters, editorial articles, or meta-analysis, and (iii) incomplete data regarding genotype distribution in cases and controls.

### Data extraction

Two researchers independently extracted data from each included study and any discrepancies were resolved through discussion. The following information was extracted from each study: first author, year of publication, disease categories, age of controls, genotyping method, sample size of cases and controls, genotype distribution in cases and controls.

### Quality evaluation of the included studies

The methodological quality of included studies was independently evaluated by two researchers according to the Newcastle–Ottawa Scale (NOS) [8]. The NOS quality score ranges from 0 to 9 stars, which is categorized based on three criteria: selection, comparability, and exposure assessment. Scores equal to or greater than 7 indicated high methodological quality; a score below 7 indicated the study should be considered ‘low quality’. Discrepancies were resolved through discussion.

### Statistical analysis

The Hardy–Weinberg equilibrium (HWE) was calculated in the control groups using the chi-squared test, with *P*<0.05 considered as a deviation. Dichotomous data are presented as OR with 95% CI. Heterogeneity was assessed using the *Q* test and quantited by the *I*^2^ test. If there was no heterogeneity (*P*>0.1 or *I*^2^ < 50%), a fixed-effects model (Mantel–Haenszel method) was used to estimate the pooled OR; otherwise, a random-effects model (Mantel–Haenszel method) was utilized. For the PvuII -397T/C polymorphism, the allele (C compared with T), dominant (CC+CT compared with TT), recessive (CC compared with TT+CT), homozygous (CC compared with TT), and heterozygous model (CC compared with CT) were evaluated. For the XbaI -351A/G polymorphism, the allele (G compared with A), dominant (GG+GA compared with AA), recessive (GG compared with AG+AA), homozygous (GG compared with AA), and heterozygous model (GG compared with GA) were evaluated. The *Z* test was conducted to estimate the statistical significance of summary results, with *P*<0.05 indicating statistical significance. Sensitivity analysis was conducted to assess the stability and quality of the pooled results. The Egger’s test was conducted to evaluate publication bias and in order to assess the potential impact of publication bias on the OR estimate, we estimated an OR adjusted for publication bias using the trim-and-fill method, which imputes potentially missing studies to achieve symmetry in the funnel plot [9]; *P*<0.05 indicated statistical significance. All statistical analysis was performed using Stata 12.0 software (Stata Corporation, College Station, TX, U.S.A.). Statistical power analysis was performed using Power and Precision V4 software (free download available at http://www.power-analysis.com/) to verify whether the included studies and meta-analysis could offer adequate power (≥80%).

## Results

### Characteristics of the included studies

**Figure 1:**
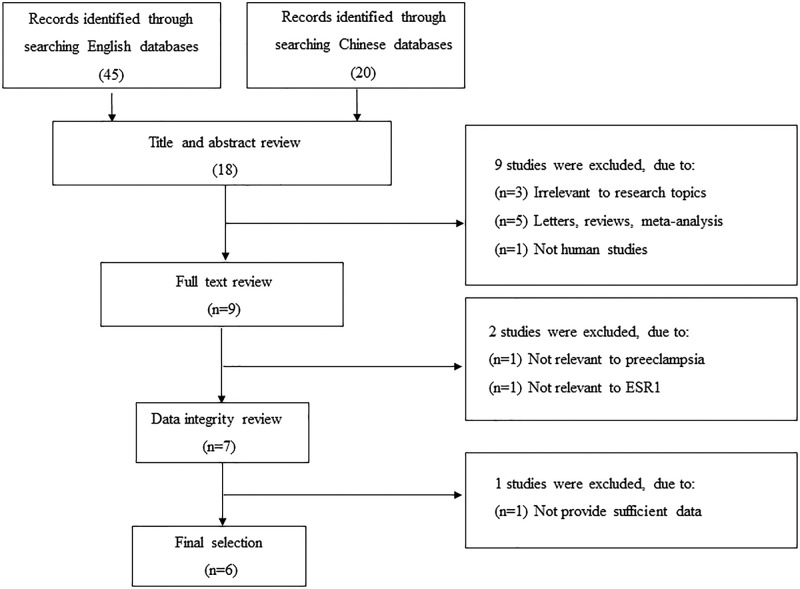
The selection process of eligible articles

The selection process of eligible articles is shown in Figure 1. The search strategy identified 65 potentially relevant articles. Based on the inclusion and exclusion criteria, six articles [10–15] (consisting of seven studies, with one article considered as two separate studies with two different subpopulations) were included in the meta-analysis. Of these seven studies: seven studies assessed the association between the ESR1 -397T/C PvuII polymorphism and severe pre-eclampsia susceptibility, five studies assessed the ESR1 -397T/C PvuII polymorphism and mild pre-eclampsia susceptibility, four studies assessed the ESR1 -351A/G XbaI polymorphism and severe pre-eclampsia susceptibility, and three studies assessed the ESR1 -351A/G XbaI polymorphism and mild pre-eclampsia susceptibility. The corresponding characteristics are shown in Table 1.

**Table 1:** Main characteristics of all studies included in the meta-analysis

Author	Year	Country/ ethnicity	Disease categories	Age (controls)	Genotyping method	Sample size (case/control)	Cases			Controls			*P* HWE	NOS score
							CC	CT	TT	CC	CT	TT		
-397T/C: PvuII restriction site (rs2234693)														
Severe pre-eclampsia														
Salimi S.	2017	Iran	Severe	26.5 ± 6.3	PCR-RFLP	192/186	9	26	15	42	94	50	0.86	7
El-Beshbishy H.A.	2015	Saudi	NA	24 ± 2.9	PCR-RFLP	97/94	21	52	24	20	44	30	0.61	7
Groten T.	2014	Caucasian	Severe	NA	PCR	27/175	5	14	14	37	74	64	0.08	7
Groten T.	2014	African	Severe	NA	PCR	16/134	3	10	3	24	78	32	0.05	7
Zhang J.	2009	Chinese Han	Severe	28.71 ± 3.60	PCR	140/236	48	66	26	78	113	45	0.72	8
Molvarec A.	2006	Caucasian	Severe	28 (25–31)	PCR	119/103	26	56	37	18	53	32	0.62	8
Tempfer C.B.	2004	Caucasian	Severe	29 (21–43)	PCR	24/24	4	14	6	4	12	8	0.89	7
Mild pre-eclampsia														
Salimi S.	2017	Iran	Mild	26.5 ± 6.3	PCR-RFLP	142/186	28	81	33	42	94	50	0.86	7
El-Beshbishy H.A.	2015	Saudi	NA	24 ± 2.9	PCR-RFLP	97/94	21	52	24	20	44	30	0.61	7
Groten T.	2014	Caucasian	Mild	NA	PCR	47/175	8	28	28	37	74	64	0.08	7
Groten T.	2014	African	Mild	NA	PCR	65/13	16	35	14	24	78	32	0.05	7
Zhang J.	2009	Chinese Han	Mild	28.71 ± 3.60	PCR	64/236	24	28	12	78	113	45	0.72	8

Author	Year	Country		Age (cases)	Genotyping method	Sample size (case/control)	Cases			Controls			P HWE	NOS score
							GG	AG	AA	GG	AG	AA		
-351A/G: XbaI restriction site (rs9340799)														
**Severe pre-eclampsia**														
Salimi S.	2017	Iran	Severe	26.5 ± 6.3	PCR-RFLP	192/186	14	17	19	19	98	69	0.06	7
El-Beshbishy H.A.	2015	Saudi	NA	24 ± 2.9	PCR-RFLP	97/94	13	54	30	10	58	26	0.01	7
Molvarec A.	2006	Caucasian	Severe	28 (25–31)	PCR	119/103	19	62	38	14	64	25	0.009	8
Zhang J.	2009	Chinese Han	Severe	28.71 ± 3.60	PCR	140/236	7	51	82	8	77	151	0.63	8
**Mild pre-eclampsia**														
Salimi S.	2017	Iran	Mild	26.5 ± 6.3	PCR-RFLP	142/186	13	82	47	19	98	69	0.06	7
El-Beshbishy H.A.	2015	Saudi	NA	24 ± 2.9	PCR-RFLP	97/94	13	54	30	10	58	26	0.01	7
Zhang J.	2009	Chinese Han	Mild	28.71 ± 3.60	PCR	64/236	3	25	36	8	77	151	0.63	8

Abbreviations: NA, not available; RFLP-PCR, restriction fragment length polymorphism PCR.

### Meta-analysis of the ESR1 -397T/C PvuII polymorphism and severe pre-eclampsia

The seven selected studies [10–15], which included 479 cases and 952 controls, were used to determine the association between the ESR1 -397T/C PvuII polymorphism and severe pre-eclampsia susceptibility. A summary of meta-analysis for the association between the ESR1 -397T/C PvuII polymorphism and severe pre-eclampsia is shown in Table 2. There was no association between the -397T/C polymorphism in the *ESR1* gene and susceptibility to severe pre-eclampsia.

**Table 2:** Meta-analysis of associations between the ESR1 gene polymorphism and severe and mild preeclampsia

polymorphism	Comparison	No. of	Test of associations		Test of heterogeneity		Model	Egger’s test	Sensitivity analysis	
		Studies	OR (95%CI)	*P* value	*I*^2^(%)	*P* value		*P* value	OR (95%CI)	*P* value
-397 T/C PvuII (rs2234693)										
Severe preeclampsia										
	C vs. T	7	1.02(0.87,1.21)	0.796	0	0.892	F	0.880	0.99(0.83,1.20)	0.960
	CC+CT vs. TT	7	1.05(0.81,1.37)	0.713	0	0.890	F	0.520	0.99(0.74,1.32)	0.917
	CC vs. TT+CT	7	1.01(0.76,1.33)	0.963	0	0.935	F	0.438	1.01(0.74,1.35)	0.982
	CC vs. TT	7	1.04(0.74,1.45)	0.828	0	0.900	F	0.874	0.99(0.68,1.43)	0.953
	CC vs. CT	7	0.99(0.74,1.33)	0.954	0	0.949	F	0.325	1.01(0.74,1.39)	0.941
Mild preeclampsia										
	C vs. T	5	1.02(0.86,1.21)	0.783	0	0.426	F	0.919	0.99(0.83,1.20)	0.967
	CC+CT vs. TT	5	1.08(0.83,1.43)	0.562	0	0.627	F	0.996	1.02(0.75,1.38)	0.907
	CC vs. TT+CT	5	0.98(0.74,1.30)	0.884	7	0.367	F	0.696	0.97(0.71,1.33)	0.850
	CC vs. TT	5	1.03(0.73,1.45)	0.888	0	0.431	F	0.888	0.97(0.66,1.43)	0.878
	CC vs. CT	5	0.95(0.70,1.29)	0.738	0	0.416	F	0.811	0.93(0.69,1.34)	0.822
-351 A/G XbaI (rs9340799)										
Severe preeclampsia										
	G vs. A	4	1.10(0.91,1.34)	0.331	0.4	0.390	F	0.636	1.14(0.91,1.43)	0.255
	GG+GA vs. AA	4	0.97(0.74,1.28)	0.846	5.8	0.407	F	0.205	1.01(0.74,1.36)	0.979
	GG vs. AA+GA	4	1.67(1.10,2.55)	0.017^a^	30.6	0.228	F	0.808	1.81(1.12,2.93)	0.016^1^
	GG vs. AA	4	1.43(0.90,2.28)	0.125	14.2	0.321	F	0.925	1.54(0.91,2.60)	0.106
	GG vs. GA	4	1.81(1.17,2.82)	0.008^a^	38	0.184	F	0.858	1.97(1.19,3.27)	0.008^1^
Mild preeclampsia										
	G vs. A	3	1.03(0.83,1.28)	0.764	0	0.563	F	0.337	1.04(0.81,1.35)	0.747
	GG+GA vs. AA	3	0.90(0.67,1.22)	0.494	35.1	0.214	F	0.483	0.91(0.65,1.29)	0.613
	GG vs. AA+GA	3	1.13(0.67,1.91)	0.647	0	0.545	F	0.212	1.04(0.54,2.01)	0.897
	GG vs. AA	3	1.12(0.64,1.97)	0.697	0	0.858	F	0.047	1.11(0.56,2.23)	0.759
	GG vs. GA	3	1.15(0.67,1.96)	0.619	14.3	0.311	F	0.202	1.03(0.52,2.02)	0.941

a *P*<0.05.

Abbreviations: OR: odds ratio; 95% CI: 95% confidence interval; F: fixed effects model; R: random effects model; No.: number.

### Meta-analysis of the ESR1 -397T/C PvuII polymorphism and mild pre-eclampsia

In total, five studies [10,11,14,15], containing 432 cases and 825 controls, identified an association between the ESR1 -397T/C PvuII polymorphism and mild pre-eclampsia susceptibility. A summary of meta-analysis for the association between the ESR1 -397T/C PvuII polymorphism and mild pre-eclampsia is shown in Table 2. We did not find a significant association between the ESR1 -397T/C PvuII polymorphism and mild pre-eclampsia in allele frequency and genotype distribution between cases and controls.

### Meta-analysis of the ESR1 -351A/G XbaI polymorphism and severe pre-eclampsia

**Figure 2:**
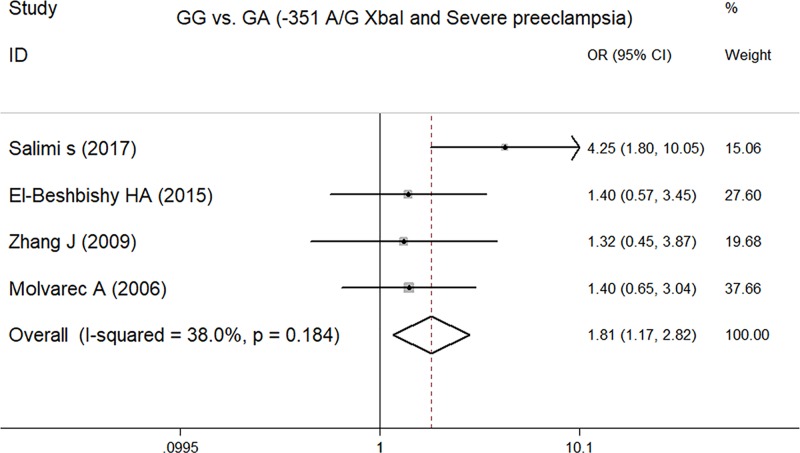
Forest plot of GG compared with GA (-351 AG XbaI and severe pre-eclampsia)

**Figure 3:**
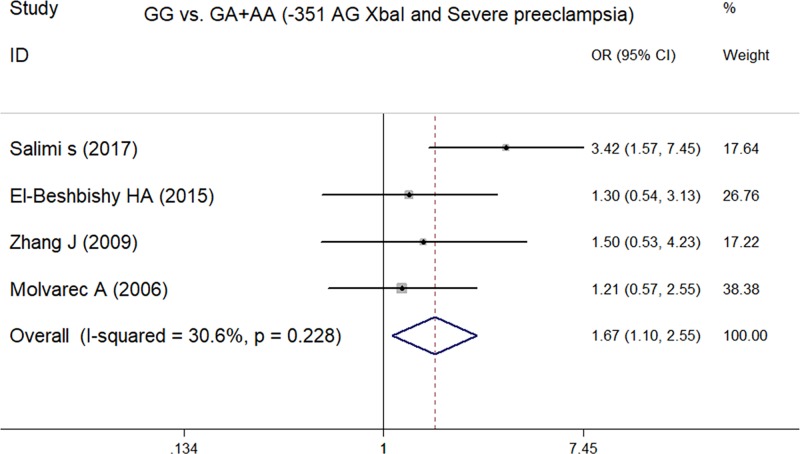
Forest plot of GG compared with GA+AA (-351 AG XbaI and severe pre-eclampsia)

In total, we identified four studies [10–12,14], containing 406 cases and 619 controls, that were used to assess the effect of the ESR1 -351A/G XbaI polymorphism on the susceptibility to severe pre-eclampsia. A summary of meta-analysis for the association between the ESR1 -351A/G XbaI polymorphism and severe pre-eclampsia is shown in Table 2. There were significant associations for both GG compared with AA+GA (OR = 1.67, 95% CI = 1.10–2.55, *P*=0.017) (Figure 2) and GG compared with GA genotype (OR = 1.81, 95% CI = 1.17–2.82, *P*=0.008) (Figure 3); other comparisons failed to identify any significant association. This indicates that the GG genotype of the ESR1 XbaI polymorphism could be a genetic risk factor for severe pre-eclampsia susceptibility.

### Meta-analysis of the ESR1 -351A/G XbaI polymorphism and mild pre-eclampsia

In total, we identified three studies [10,11,14], containing 303 cases and 616 controls, that were used to assess the effect of the ESR1 -351A/G XbaI polymorphism on mild pre-eclampsia susceptibility. A summary of meta-analysis for the association between the ESR1 -351A/G XbaI polymorphism and mild pre-eclampsia is shown in Table 2. We did not find a significant association between the ESR1 -351A/G XbaI polymorphism and mild pre-eclampsia in allele frequency and genotype distribution between cases and controls.

### Sensitivity analysis

In one study, it is unclear whether the pre-eclampsia patients were mildly or severely affected [14], so we performed sensitivity analysis excluding this study to check the robustness of our result. This study was omitted to evaluate the influence on the combined OR. The results show that the corresponding pooled OR was not qualitatively altered (Table 2), indicating that the pooled analysis was stable and reliable. Moreover, the genotype distribution of the controls in two studies [12,14] significantly deviated from HWE in this meta-analysis. We also performed sensitivity analysis excluding the HWE-violating studies to check the robustness of our results. Our findings show that before excluding the two studies that deviated from HWE [12,14], the GG genotype increased the risk of severe pre-eclampsia. However, after excluding the two studies, the results were materially altered and the effect of the GG genotype on the risk of severe pre-eclampsia was not significant.

### Publication bias

To identify publication bias, the Egger’s test was performed on each model of the ESR1 gene PvuII and XbaI polymorphisms. The results suggest that there was evidence for publication bias in the GG compared with AA model of the -351A/G XbaI (rs9340799) polymorphism and mild pre-eclampsia, as indicated by Egger’s asymmetry test (*P*=0.047) (Table 2). Using the trim-and-fill method, four additional artificial studies were included in the meta-analysis to generate a symmetric funnel plot. This correction for publication bias yielded an OR of 1.01 (95% CI = 0.63–1.59; *P*=0.921), however, the presence of publication bias did not influence the OR estimate and overall result (OR = 1.12, 95% CI = 0.64–1.97, *P*=0.697). The subsequent trim-and-fill method showed that publication bias had little impact on the stability of the results.

### Statistical power analysis

We adopted the statistical power analysis to reassess the available data when an α of 0.05 and β of 0.2 were assigned. The power of each eligible study of the association between the ESR1 -351A/G Xbal polymorphism and severe and mild pre-eclampsia susceptibility ranged from 5% to 89%, 5% to 45%, respectively. In the meta-analysis of the association between the ESR1 -351A/G XbaI polymorphism and severe pre-eclampsia susceptibility, the power analysis suggests that a power of 57% was required to detect a pooled OR for the GG compared with GA model, and 72% for a pooled OR for the GG compared with AA+GA model. The specific power values are summarized in Table 3.

**Table 3:** Statistical power analysis of eligible studies and meta-analyses of ESR1 -351 A/G XbaI polymorphism and severe and mild pre-eclampsia susceptibility

Study	Comparison	Salimi S.	El-Beshbishy H.A.	Zhang J.	Molvarec A.	Overall
-351 A/G and severe pre-eclampsia						
	G vs. A	28%	5%	16%	10%	46%
	GG+GA vs. AA	5%	7%	16%	26%	10%
	GG vs. AA+GA	83%	10%	16%	7%	72%
	GG vs. AA	56%	5%	15%	6%	63%
	GG vs. GA	89%	9%	9%	12%	57%
-351 A/G and mild pre-eclampsia						
	G vs. A	6%	22%	5%	25%	6%
	GG+GA vs. AA	12%	42%	7%	14%	12%
	GG vs. AA+GA	6%	22%	10%	26%	6%
	GG vs. AA	5%	10%	5%	45%	5%
	GG vs. GA	7%	26%	9%	39%	7%

## Discussion

The familial nature of pre-eclampsia has been known for a number of years, and the identification of novel susceptibility genes is one of many strategies aimed at fully elucidating the underlying biological pathogenetic mechanisms [16]. The gene encoding ESR1 is located on the 6q25.1 chromosome and includes eight exons and seven introns, encoding a protein of 595 amino acids with a molecular size of 66 kDa [17]. A multitude of variations in the ESR1 gene have been identified, which may influence ESR1 protein structure or activity. The most common polymorphisms are rs2234693 (T>C: PvuII) and rs9340799 (A>G: XbaI), which are located in intron 1 of the *ESR1* gene. The PvuII, T397C polymorphism occurs due to T>C transition in intron 1; while the XbaI, G351A polymorphism is a G>A transition located 50 bp from the PvuII polymorphic site [18]. For the ESR1 PvuII gene polymorphism, it has been demonstrated that the C allele forms part of a functional binding site for the B-myb transcription factor and it acts as an intragenic enhancer [19]. As estrogen is believed to be involved in up-regulating the transcription of myb [20], the presence of a PvuII site corresponding to the T allele may lead to decreased ESR1 expression, and therefore the effects of estrogen mediated by ESR1 may be reduced, resulting in a relative estrogen deficit. The XbaI polymorphism might also have functional importance that remains unclear [12]. Interpretation of genetic studies involving pre-eclampsia is hindered by differences in definition of the disease, size of study population, diverse ethnicity between studies, and ethnic mixing within studies [16]. Furthermore, environmental factors may also affect an individual’s susceptibility to the development of pre-eclampsia [11]. Further large-scale studies with different ethnicities are required to gain insight and explore the mechanism of ESR1 gene polymorphism in pre-eclampsia.

Our meta-analysis suggests that the GG genotype of the ESR1 XbaI polymorphism could be a genetic risk factor for severe pre-eclampsia susceptibility. As HWE is a surrogate to assess study quality, the chi-squared test was conducted to check whether genotype distributions in controls conformed to the HWE rule. However, the genotype distribution of the controls in two studies [12,14] significantly deviated from HWE in this meta-analysis. Sensitivity analysis was performed by excluding the HWE-violating studies to check the robustness of our results. Our findings show that before excluding the two studies that deviated from HWE [12,14], the GG genotype increased the risk of severe pre-eclampsia. However, upon excluding the two studies, the results were materially altered and the effect of the GG genotype on the risk of severe pre-eclampsia was not significant. The deviation from HWE could be due to population stratification, non-random mating, genotyping error, genetic drifting, chance, and selection bias. In the case of meta-analysis, it is unclear which factor is responsible due to insufficient data. Sensitivity analysis including and excluding the HWE-violating studies was recommended [21]; consequently, the results of this meta-analysis should be interpreted with caution. Minelli et al. [22] found no evidence of a strong association between departures from HWE and estimating the genetic effect.

The meta-analysis was a powerful way to effectively increase the sample size to provide a more valid pooled estimate. However, the highest statistical power of the pooled result on the overall meta-analysis of the ESR1 -351A/G XbaI polymorphism and severe and mild pre-eclampsia susceptibility was found to be 72 and 12%, respectively, which are less than 80%, i.e. the powers of our findings were unsatisfactory. Owing to the low statistical power, the results may not be sufficiently robust, and any conclusions should be interpreted cautiously. Statistical power depends upon the population effect size, number of studies, and average sample size. As the pooled sample size (only three or four studies) is not large enough, the statistical power to estimate the effect of this locus may be limited, and thus further large-scale and high-quality studies are necessary to validate our findings.

There was evidence of publication bias in the GG compared with AA model of the -351A/G XbaI (rs9340799) polymorphism and mild pre-eclampsia, however, this did not impact the combined OR estimate (Duval and Tweedie’s trim-and-fill adjusted OR). The subsequent trim-and-fill method showed that publication bias had little influence on the stability of the results. Publication bias of our meta-analysis was attributed to the limited availability of published results, as the number of publications included in our meta-analysis was relatively small.

The present meta-analysis has some limitations that should be discussed. First, only four studies assessed the ESR1 -351A/G XbaI polymorphism and severe pre-eclampsia susceptibility, and three studies assessed the ESR1 -351A/G XbaI polymorphism and mild pre-eclampsia susceptibility. Hence, the limited study number and small sample size may not offer sufficient statistical power to investigate the association between the ESR1 polymorphisms and the risk of severe or mild pre-eclampsia. As the statistical power may be limited, more large-scale and high-quality studies will be required to evaluate the risk of pre-eclampsia in different ethnic groups and validate the meta-analysis. Second, all the included studies are case–control and all had the limitation of being observational studies, including selection bias and unmeasured confounders. Third, our meta-analysis did not investigate the genetic haplotypes of severe or mild pre-eclampsia, and only single locus SNP -397T/C PvuII or -351A/G XbaI in the *ESR1* gene were analyzed in the present study. It therefore remains unclear whether additional genetic variants contribute to this gene; investigating haplotypes reveal more information regarding the genetic causes of diseases than genotypes and hence would be more influential than single SNPs. Fourth, including only English and Chinese articles may produce publication bias and more eligible studies could be captured if the search was extended. The selection bias caused by language restriction may reduce the robustness of our meta-analysis [23,24]. Finally, environmental factors may affect the development of pre-eclampsia; however, we did not consider environmental factors, such as living habits and diets, or potential gene–environment interactions that might lead to bias in the results.

## Conclusion

In conclusion, our meta-analysis suggests that the GG genotype of the ESR1 Xbal polymorphism may be a genetic risk factor for severe pre-eclampsia susceptibility. However, there is no association between the ESR1 gene Pvull and Xbal promoter polymorphism and the risk of mild pre-eclampsia. Due to the limitations mentioned above, well-designed and larger scale studies are necessary to validate our results, and such investigations may explore the roles of the ESR1 XbaI gene polymorphism in the pathogenesis of severe pre-eclampsia.

## Abbrevations

CI: confidence interval
CNKI: China National Knowledge Infrastructure
ESR1: estrogen receptor α
HWE: Hardy–Weinberg equilibrium
NOS: Newcastle–Ottawa Scale
OR: odds ratio

## References

[1] Roberts J.M. and Cooper D.W. (2001) Pathogenesis and genetics of pre-eclampsia. Lancet 357, 53–56 10.1016/S0140-6736(00)03577-7 11197372

[2] Sibai B., Dekker G. and Kupferminc M. (2005) Pre-eclampsia. Lancet 365, 785–799 10.1016/S0140-6736(05)71003-5 15733721

[3] Parker M.G., Arbuckle N., Dauvois S., Danielian P. and White R. (1993) Structure and function of the estrogen receptor. Ann. N.Y. Acad. Sci. 684, 119–126 10.1111/j.1749-6632.1993.tb32276.x 8317825

[4] Mendelsohn M.E. and Karas R.H. (1999) The protective effects of estrogen on the cardiovascular system. N. Engl. J. Med. 340, 1801–1811 10.1056/NEJM199906103402306 10362825

[5] Lehrer S., Rabin J., Kalir T. and Schachter B.S. (1993) Estrogen receptor variant and hypertension in women. Hypertension 21, 439–441 10.1161/01.HYP.21.4.439 8458645

[6] Lehrer S., Sanchez M., Song H.K. (1990) Oestrogen receptor B-region polymorphism and spontaneous abortion in women with breast cancer. Lancet 335, 622–624 10.1016/0140-6736(90)90410-7 1969015

[7] Smith E.P., Boyd J., Frank G.R. (1994) Estrogen resistance caused by a mutation in the estrogen-receptor gene in a man. N. Engl. J. Med. 331, 1056–1061 10.1056/NEJM199410203311604 8090165

[8] Stang A. (2010) Critical evaluation of the Newcastle-Ottawa scale for the assessment of the quality of nonrandomized studies in meta-analyses. Eur. J. Epidemiol. 25, 603–605 10.1007/s10654-010-9491-z 20652370

[9] Duval S. and Tweedie R. (2000) Trim and fill: a simple funnel-plot-based method of testing and adjusting for publication bias in meta-analysis. Biometrics 56, 455–463 10.1111/j.0006-341X.2000.00455.x 10877304

[10] Salimi S., Farajian-Mashhadi F., Tabatabaei E., Shahrakipour M., Yaghmaei M. and Mokhtari M. (2017) Estrogen receptor alpha XbaI GG genotype was associated with severe preeclampsia. Clin. Exp. Hypertens. 39, 220–224 10.1080/10641963.2016.1235182 28448182

[11] Zhang J., Bai H., Liu X. (2009) Genotype distribution of estrogen receptor alpha polymorphisms in pregnant women from healthy and preeclampsia populations and its relation to blood pressure levels. Clin. Chem. Lab. Med. 47, 391–397 10.1515/CCLM.2009.096 19284296

[12] Molvarec A., Ver A., Fekete A. (2007) Association between estrogen receptor alpha (ESR1) gene polymorphisms and severe preeclampsia. Hypertens. Res. 30, 205–211 10.1291/hypres.30.205 17510501

[13] Tempfer C.B., Jirecek S., Riener E.K. (2004) Polymorphisms of thrombophilic and vasoactive genes and severe preeclampsia: a pilot study. J. Soc. Gynecol. Investig. 11, 227–231 10.1016/j.jsgi.2003.12.002 15120696

[14] El-Beshbishy H.A., Tawfeek M.A., Al-Azhary N.M. (2015) Estrogen receptor alpha (ESR1) gene polymorphisms in pre-eclamptic Saudi patients. Pakistan J. Med. Sci. 31, 880–885 10.12669/pjms.314.7541 PMC4590376 26430422

[15] Groten T., Schleussner E., Lehmann T. (2014) eNOSI4 and EPHX1 polymorphisms affect maternal susceptibility to preeclampsia: analysis of five polymorphisms predisposing to cardiovascular disease in 279 Caucasian and 241 African women. Arch. Gynecol. Obstet. 289, 581–593 10.1007/s00404-013-2991-9 24013430

[16] Mutze S., Rudnik-Schoneborn S., Zerres K. and Rath W. (2008) Genes and the preeclampsia syndrome. J. Perinat. Med. 36, 38–58 10.1515/JPM.2008.004 18184097

[17] Rigo J. Jr, Boze T., Derzsy Z. (2006) Family history of early-onset cardiovascular disorders is associated with a higher risk of severe preeclampsia. Eur. J. Obstet. Gynecol. Reprod. Biol. 128, 148–151 10.1016/j.ejogrb.2006.02.019 16678332

[18] Roberts J.M. and Gammill H. (2005) Pre-eclampsia and cardiovascular disease in later life. Lancet 366, 961–962 10.1016/S0140-6736(05)67349-7 16168757

[19] Herrington D.M., Howard T.D., Brosnihan K.B. (2002) Common estrogen receptor polymorphism augments effects of hormone replacement therapy on E-selectin but not C-reactive protein. Circulation 105, 1879–1882 10.1161/01.CIR.0000016173.98826.88 11997270

[20] Swartz C.D., Afshari C.A., Yu L., Hall K.E. and Dixon D. (2005) Estrogen-induced changes in IGF-I, Myb family and MAP kinase pathway genes in human uterine leiomyoma and normal uterine smooth muscle cell lines. Mol. Hum. Reprod. 11, 441–450 10.1093/molehr/gah174 15879465

[21] Thakkinstian A., McElduff P., D’Este C., Duffy D. and Attia J. (2005) A method for meta-analysis of molecular association studies. Stat. Med. 24, 1291–1306 10.1002/sim.2010 15568190

[22] Minelli C., Thompson J.R., Abrams K.R., Thakkinstian A. and Attia J. (2008) How should we use information about HWE in the meta-analyses of genetic association studies? Int. J. Epidemiol. 37, 136–146 10.1093/ije/dym234 18037675

[23] Egger M., Davey Smith G., Schneider M. and Minder C. (1997) Bias in meta-analysis detected by a simple, graphical test. BMJ 315, 629–634 10.1136/bmj.315.7109.629 9310563 PMC2127453

[24] Egger M., Zellweger-Zahner T., Schneider M., Junker C., Lengeler C. and Antes G. (1997) Language bias in randomised controlled trials published in English and German. Lancet 350, 326–329 10.1016/S0140-6736(97)02419-7 9251637

